# *Besnoitia besnoiti*-Induced Neutrophil Extracellular Traps (NETs): Metabolic Signature, Signaling Pathways, Receptors and Implications on Pathogenesis

**DOI:** 10.3390/ani15223326

**Published:** 2025-11-18

**Authors:** Nicolás Turra, Iván Conejeros, Carlos Hermosilla, Rafael Agustín Burgos, Anja Taubert

**Affiliations:** 1 Institute of Pharmacology and Morphophysiology, Faculty of Veterinary Sciences, Austral University of Chile, Valdivia 509000, Chile; rburgos1@uach.cl; 2 Institute of Parasitology, Faculty of Veterinary Medicine, Justus Liebig University Giessen, 35392 Giessen, Germany; ivan.conejeros@uni-giessen.de (I.C.); anja.taubert@vetmed.uni-giessen.de (A.T.)

**Keywords:** *Besnoitia besnoiti*, neutrophil extracellular traps (NETs), bovine NETosis, cattle, innate immunity

## Abstract

*Besnoitia besnoiti* is an apicomplexan parasite responsible for bovine besnoitiosis, a debilitating disease in cattle resulting in local and systemic clinical signs with detrimental effects on reproductive performance and productivity in livestock. Fast-replicating tachyzoites and slowly proliferating bradyzoites elicit an excessive host innate immune response, mainly by activated polymorphonuclear neutrophils (PMN), which extrude neutrophil extracellular traps (NETs) as a defense mechanism. These PMN-derived structures, composed principally of DNA, histones, and peptides, play a crucial role not only in parasite entrapment but also in NET-associated endothelial damage, thereby most likely contributing to the pathogenesis of this neglected cattle parasitosis. Uncontrolled production of NETs or their inadequate removal may perpetuate an inflammatory environment in the vasculature and epidermis. Thus, novel alternative treatment of animals with chronic bovine besnoitiosis displaying severe clinical manifestations such as hyperkeratosis, vulvovaginitis and orchitis, could be considered for future study to either hampering NETs release or reducing NETs concentrations in affected tissues. Since effective treatments and control strategies for bovine besnoitiosis do not yet exist, this review serves as a guide for further research on the metabolic signature, signaling pathways, receptors, and pathogenesis of *B. besnoiti*-triggered NETs formation, providing insights into potential therapeutic approaches to avoid excessive NETs extrusion.

## Simple Summary

Bovine besnoitiosis is a neglected debilitating parasitic disease of cattle caused by the apicomplexan parasite *Besnoitia besnoiti*, resulting in significant economic losses for livestock producers. Due to expansion of the disease into previous non-endemic European countries as well as the lack of effective treatments and control strategies to manage it, there is a need to elucidate early host innate immune reactions during acute- and chronic bovine besnoitiosis. Polymorphonuclear neutrophils (PMN) are the first leukocytes recruited to the site of infection. Consequently, an important defense mechanism displayed by bovine PMN to combat invading pathogens is the extrusion of neutrophil extracellular traps (NETs). However, acute and chronic *B. besnoiti* parasitic stages (i.e., tachyzoites and bradyzoites) elicit an excessive host innate immune response, leading to possible NET-associated inflammation and tissue injury, thereby contributing to the pathogenesis of this cattle parasitosis. Thus, to provide insights into potential therapeutic approaches to avoid excessive NETs extrusion in vivo, the present review will focus on the metabolic signature, signaling pathways, receptors, and pathogenesis of *B. besnoiti*-triggered bovine NETs formation.

## 1. Introduction

### 1.1. The Apicomplexan Parasite Besnoitia Besnoiti: Taxonomy, Pathogeny and Clinical Signs

*Besnoitia besnoiti* is an obligatory intracellular protozoan parasite belonging to the subfamily Toxoplasmatinae (family Sarcocystidae), which is part of the cyst-forming coccidia clade within the phylum Alveolata (subphylum Apicomplexa) [1,2,3]. Accordingly, *B. besnoiti* forms a phylogenetic taxon closely related to *Toxoplasma gondii* and *Neospora caninum* [3,4], sharing certain common morphological and biological features. In contrast to the genus *Toxoplasma*, there is no evidence that the genus *Besnoitia* is anthropozoonotic [5]. Currently, *Besnoitia* species have been described [6,7], comprising six species affecting mainly small mammals (rodents), lagomorpha (rabbits), marsupials (opossums) and lacertids (lizards) (*Besnoitia darlingi, Besnoitia jellisoni, Besnoitia wallacei, Besnoitia oryctofelisi, Besnoitia akodoni* and *Besnoitia neotomofelis*) acting as intermediate hosts, and having felids as definitive hosts [5,8]. Two other species have been described in ruminants *Besnoitia caprae* (goats) [9] and *Besnoitia tarandi* (reindeer) [10]. Only one species, *Besnoitia bennetti,* has been reported to affect domestic and wild equids [6,11,12].

Bovine besnoitiosis has gained increased attention in the last two decades owing to its negative impact on cattle industry and re-emergence in previously non-endemic countries [1,10,13,14]. *B. besnoiti* is the only etiological agent of the disease [1,6,15,16] resulting in a rather chronic debilitating parasitosis characterized by both local- and systemic clinical signs of varying severity [4,10] thereby leading to significant economic losses in both dairy- and beef cattle industry, which are evidenced by a marked decrease in productivity and reproduction in affected herds [6,16]. Currently, international animal trade is considered the main risk factor contributing to the spread of *B. besnoiti* infections in Europe [4,13,17]. Due to this epidemiological spread, the European Food Safety Authority (EFSA) has classified bovine besnoitiosis as an emerging disease within the European Union in 2010 [13,14,18].

Concerning the biology, a heteroxenous life cycle has been proposed for *B. besnoiti* involving two hosts [16], that is, an unknown carnivorous definitive host (DH), where *B. besnoiti* sexual stages such as male micro- and female macrogamonts occur within intestinal epithelial cells (IEC), resulting in oocyst production, and the bovine intermediate host (IH), where two parasitic stages occur, namely the fast-replicating tachyzoites and the slowly replicating bradyzoites [19,20]. Once the parasite is established in the bovine IH, an estimated infection time of 11–14 days before the onset of clinical symptoms has been considered [16]. The start of infection is marked by an acute phase that can last from 3 to 10 days [14], when tachyzoites undergo rapid asexual intracellular proliferation, mainly in endothelial cells of vascular and lymphatic vessels, as well as professional phagocytes, including macrophages, monocytes, and polymorphonuclear neutrophils (PMN) [1,21,22]. Fast-replicating *B. besnoiti* tachyzoites generate vasculitis, hyperplasia, lymphadenopathies, thrombosis, and necrosis of venules and arterioles, leading to increased vascular permeability and causing subcutaneous edema, mainly in the ventral areas of the body as well as in the limbs [10,16]. Notably, this clinical stage is also known as anasarca in bovine besnoitiosis [14,19]. Other non-specific clinical signs during the acute phase include fever, depression, tachycardia, tachypnea, mucosal congestion, nasal and ocular discharge, stiffness of gait, necrotizing orchitis in males, anorexia, necrotizing mastitis/thelitis in females, and weight loss [19,23]. Conversely, in the chronic phase, also called the scleroderma stage, tachyzoites differentiate into bradyzoites, which proliferate slowly and are encapsulated in large tissue cysts with diameters of 200–600 µm in vivo [5,10]. These large cysts are formed within the epidermis, subcutaneous tissue, muscle fascia, nasal and genital mucosa, and sclera conjunctiva, and endothelium of blood vessels, but mainly in connective tissues [5,14]. Dermal tissue cysts are responsible for skin lesions, resulting in thickening, hardening, and wrinkling of the epidermis (i.e., “elephant skin”), hyperkeratosis, skin nodules, and alopecia [4], as well as testicular atrophy, photophobia, and arthritis [14].

Due to the lack of effective chemotherapy treatments, as well as the difficulty in implementing satisfactory control and prevention strategies, there is still a need to better study interactions between professional phagocytes, endothelium, epithelium, and *B. besnoiti* to provide novel insights on early host innate reactions such as reported neutrophil extracellular traps (NETs) formation [24,25], deciphering either its protective or adverse role by increasing chronic subcutaneous inflammation and endothelium damage [26]. Thus, inhibitors of *B. besnoiti*-triggered NETosis associated adverse effects on tissue/vessels might be considered as alternative treatment for besnoitiosis in future as already reported for NET-associated diseases.

### 1.2. Innate Immune System and NET Formation

To combat the arrival of any infection, mammalian hosts have an innate immune system with various professional phagocyte populations displaying early non-specific and specific defense mechanisms against any invading pathogen (virus, bacteria, fungi, and parasites) and acting as the first line of defense [21]. Mammalian professional phagocytes include polymorphonuclear neutrophils (PMN), monocytes, and macrophages, as well as highly immunoreactive host epithelial and vascular/lymphatic endothelial cells covering all mucosa and vascular surfaces of the host [4,10,14]. The fastest, most mobile, and most abundant mammalian leukocytes are PMN, which are produced in the bone marrow and rapidly congregate in the infection area, despite their short lifespan, and perform diverse strategies to fight invasive pathogens [27,28,29]. Upon activation, PMN can execute various functions, including phagocytosis, degranulation, cytokine/chemokine release, secretion of extracellular vesicles (EV), and reactive oxygen species (ROS) production [28,30,31,32,33]. Additionally, another antimicrobial mechanism described in PMN to effectively combat apicomplexan parasites is the formation and release of neutrophil extracellular traps (NETs). NETs consist of network-like structures composed of DNA fibers (mainly nuclear DNA) as a backbone and decorated primarily with citrullinated histones (H1, H2A/H2B, H3, H4), followed by granular proteases and antimicrobial peptides, including neutrophil elastase (NE), myeloperoxidase (MPO), cathepsin G, leukocyte proteinase 3 (PR3), lactoferrin, gelatinase, lysozyme C, calprotectin, pentraxin, cathelicidins (LL37), and neutrophil defensins [30]. These extracellular traps (ETs) are released through a type of cell death mechanism, different from apoptosis and necrosis, called suicidal NETosis [34,35]. Classical suicidal NET formation is described as a mechanism dependent on the nicotinamide adenine dinucleotide phosphate oxidase (NOX), which forms an enzyme complex upon PMN activation by stimuli. Once NOX is activated, it leads to intracellular ROS production. ROS production modulates the enzyme MPO, whose participation is necessary for the release of serine protease NE from PMN granules. NE is then translocated to the PMN nucleus, where it strongly contributes to chromatin decondensation through histone proteolysis. Consequently, the nucleus delobulates and pores are formed in the nuclear envelop through the pore-forming protein gasdermin [36], allowing the contact of chromatin with cytoplasmic and granular proteins. Finally, the PMN cytoplasmic membrane ruptures, resulting in nuclear extrusion of DNA along with granular antimicrobial components, shaping the release of NETs [28,35,36,37]. Notably, two forms of NET release have been identified: vital NETosis and vesicular NETosis [36,38]. Nevertheless, exclusively suicidal- and vital NETosis against *B. besnoiti* and *T. gondii* have been reported in the literature [24,39]. Other closely related coccidian parasites, i.e., *N. caninum*, *Eimeria bovis*, *Eimeria ninakohlyakimovae*, *Eimeria arloingi* and *Cryptosporidium parvum* as well as euglenozoan *Trypanosoma brucei brucei* have been reported to induce suicidal NETosis [30]. Despite these reports, there is currently limited knowledge on the interactions of bovine PMN, monocytes, endothelium, and epithelium with *B. besnoiti,* despite being the first host cell types infected in vivo during the acute and chronic phases of bovine besnoitiosis [10,14,21,24,26].

### 1.3. Literature Search Strategy

This review followed a non-experimental design based on the inquiry and selection of scientific literature related to signaling pathways and metabolic processes in bovine neutrophils involved in the formation of NETs against *Besnoitia besnoiti*.

The literature search was conducted using different combinations of the keywords “Besnoitia”, “*Besnoitia besnoiti*”, “Bovine”, “Cattle”, “Extracellular traps”, “Neutrophil extracellular trap”, “Neutrophil extracellular traps”, “NETosis”, and “PMN”. We searched scientific publications available from 2004 to 2025 in the main databases PubMed, Scopus, and Web of Science (WoS) for exploration, selection, and analysis of relevant information.

Titles, abstracts, and keywords of the retrieved articles were screened, and inclusion criteria were applied to select material relevant to the main focus of this review (Table 1). The main inclusion criterion considered all scientific publications from 2004 onward—articles, review papers, and short communications—that involved the formation and release of bovine neutrophil extracellular traps in response to the cattle parasite *Besnoitia besnoiti*, as mentioned in the title and/or abstract. Additionally, some indirectly related references were included, describing NETs in non-bovine species and other parasites, in order to compare similarities and differences between host mechanisms and infectious agents.

## 2. NETs-Derived Effects on *Besnoitia besnoiti*

PMN are more heterogeneous than previously anticipated, since these cells can combat invasive pathogens by selecting different effector strategies according to the pathogen size and its motility as seen for parasitic nematodes [46,47,48] or facing a wide variety of tiny microorganisms through one defense mechanism [37]. Consistently, recent reports in the literature have shown that bovine NET formation is an important effector mechanism particularly against cyst-forming apicomplexan parasites including *T. gondii*, *N. caninum* and *B. besnoiti* among others [24,39]. Of note, monocyte-derived extracellular traps (METs) triggered by *B. besnoiti* tachyzoites have also been reported in the bovine system [21], representing the first report in the literature indicating a rather conserved and shared effector mechanism within different leukocyte populations. Although researchers have concentrated efforts on unraveling mechanisms behind NET- and MET release in response to various stimuli in the last two decades [49], there is still scarce information on molecules, receptors, signaling- and metabolic pathways linked to bovine NETosis against parasites [37,50]. The first report on *B. besnoiti*-triggered NETosis showed that the encounter of bovine PMN with vital and motile tachyzoites resulted in a strong release of NETs, thereby preventing parasitic invasion into primary bovine umbilical vein endothelial cells (BUVEC), achieving a 40% reduction in the infection rate compared to the negative control [24]. This early effector mechanism efficiently interferes not only with the tachyzoite cell invasion but also with their intracellular replication. Thus, NETosis seems to prevent further subsequent intracellular development [24] and might play a pivotal role during the acute phase of infection. Scanning electron microscopy (SEM) and immunofluorescence microscopy (IFM) analyses have revealed firm tachyzoite entrapment by *B. besnoiti*-induced NETs [24,41,42,44]. Regarding the components of *B. besnoiti*-induced NETosis, the main molecules correspond to extracellular DNA, citrullinated histones (e.g., citH3), NE, and MPO [24,26,41] which are compatible with the classic characteristics of parasite-triggered NETs as well as bacteria-mediated NET formation [34,35,51,52,53,54,55,56,57,58,59,60]. Moreover, bovine NET formation seems to be a fast cellular process carried out within minutes [24,42], as evidenced after 30 min of *B. besnoiti* tachyzoite exposure, suggesting that this defense mechanism does not exhibit an evident time dependence, since viable parasites can induce reactions of almost equal strength regardless of the incubation time [24]. Conversely, reports have described that NET induction seems to be dependent on incubation time in bovine PMN co-cultured with other closely related apicomplexan species, i.e., *N. caninum*, *E. bovis* and *C. parvum* as well as caprine PMN exposed to *E. arloingi* sporozoites [30]. NET quantification increases as the proportion of *B. besnoiti* tachyzoites against PMN enhances, indicating that NETosis is a dose-dependent process [24]. Similarly, bovine PMN release NETs against vital *N. caninum* tachyzoites in a dose-dependent manner [30]. In line with other investigations, NET release from human PMN is dependent on the number of *Leishmania* promastigotes exposed to these cells [61]. Similar results on dose dependency were obtained in bovine PMN stimulated with *E. bovis* sporozoites [30] and in ovine and bovine NET release against *T. gondii* tachyzoites [39]. Additionally, it was observed that different states of viability and integrity of *B. besnoiti* tachyzoites [i.e., viable, attenuated (via UV-irradiation), dead (via heat-inactivation) or crushed (via homogenization) tachyzoites] triggered bovine NET formation in a statistically significant manner; however, this occurred in different magnitudes when compared to non-exposed PMN [24]. These data indicate that although the amount of NETs is influenced by the viability status of *B. besnoiti* tachyzoites, NETosis is effective against all states of parasite survival. *B. besnoiti* tachyzoites induce NETosis in 6–8% of bovine PMN [41,42], which, while a small percentage within the PMN population, still managed to immobilize about one-third of the tachyzoites challenged by bovine PMN [24]. In contrast, it was estimated that 15% of bovine PMN exposed to *B. besnoiti* tachyzoites released NET at a PMN/tachyzoite ratio of 1:4 [41]. Taken together, NETosis is considered a mechanism that hinders the active invasion of *B. besnoiti* prior to infecting the host cells [24], similar to that described in bovine PMN against other apicomplexan parasites such as *N. caninum* and *T. gondii* [39]. Nevertheless, NETs do not exhibit lethal activity against successfully captured tachyzoites [24]. However, since *B. besnoiti* is an obligate intracellular parasite, it is proposed that trapping tachyzoites prior to host cell invasion could impact the outcome of bovine besnoitiosis [24]. Although NET formation was shown to be capable of trapping *B. besnoiti* tachyzoites prior to host cell invasion, it was reported that once parasites have managed to infect endothelial cells, they can also trigger NETosis in PMN adhered to the *B. besnoiti*-infected primary endothelium, as observed by scanning electron microscopy (SEM) and confirmed by DNA staining experiments [40]. Nonetheless, NET release has no direct effect on the intracellular replication of *B. besnoiti* tachyzoites, as it fails to affect the overall proliferation of the parasite despite the damage generated by NET-derived histones on infected endothelial cells [26]. Taken together, these data suggest that NET can effectively trap *B. besnoiti* tachyzoites only in direct contact, but not indirectly so far. Whether the release of NET-associated histones and pro-inflammatory molecules might not only damage the endothelium as reported by Conejeros et al. [26] but also perpetuate the pro-inflammatory status of *B. besnoiti*-infected organs requires further investigation. The same holds true for investigations focusing on effective inhibition of NETs as an alternative treatment option as this defense mechanism can become dysregulated, thereby promoting infectious-, inflammatory-, autoimmune- and neoplastic disorders in humans and animals.

## 3. Signaling Pathways Involved in Bovine NETosis Against *B. besnoiti*

Classic suicidal NETosis is described through mitogen-activated protein kinase (MAPK)-dependent pathways, also called the Raf-MEK-ERK pathway, involving the production of NADPH oxidase-derived ROS [28,30,62]. In contrast to NADPH oxidase (NOX)-dependent NETosis, another type of NET formation has been described as NOX-independent, which has been associated with substantially reduced levels of ERK1/2 activation and weak Akt activity, whereas the activation of p38 MAPK is similar in both NOX-dependent and NOX-independent pathways [30,63]. To date, *B. besnoiti*-mediated NET formation appears to depend on the activities of NOX, NE, and MPO, with the consequent production of ROS, comprising a process that can effectively prevent the active invasion of *B. besnoiti* tachyzoites into host cells [24]. The activity of these molecules was confirmed by treatments with their respective inhibitors, suggesting a significant role of their enzymatic activities for the NET development within bovine PMN exposed to *B. besnoiti* tachyzoites [24]. Thus, the relevance of NOX has been underlined in parasite-mediated NETosis, and the co-localization of NE, MPO, and histones in the DNA backbone of NET structures has been emphasized as a preserved characteristic in NET formation [30]. Notably, histone citrullination by the enzyme peptidylarginine deiminase 4 (PAD4) has not been descripted in *B. besnoiti*-induced NETosis so far [28]. PAD4-mediated histone citrullination is a mechanism that allows decondensation and unfolding of PMN nuclear DNA, which is a key NETosis event [28,64,65]. Therefore, it would be interesting to elucidate whether PAD4 plays a role in the citrullination of histones during *B. besnoiti*-induced NET formation, since there are currently no published data evaluating the role of PAD4 in the bovine system regarding NETosis. Pharmacologic or genetic suppression of PAD4 activity has been reported to attenuate disease manifestations in multiple experimental models in which NETs play a pathogenic role, including rheumatoid arthritis, systemic lupus erythematosus, atherosclerosis, and ischemia–reperfusion injury. Moreover, PAD4 appears capable of citrullinating additional protein substrates whose functional significance during NETosis remains to be determined. Therefore, the consequences of PAD4 blockade may, at least partially, be independent of histone citrullination. Further investigations employing neutrophil-specific PAD4 knockout models will be instrumental in clarifying this aspect. Nonetheless, when assessed together with other NET-associated markers (e.g., neutrophil elastase or myeloperoxidase), citrullinated histone H3 continues to serve as a reliable indicator for NET identification [28].

Of note, despite the fact that intracellular signaling pathways, such as NOX- and PAD4-mediated processes, have been linked to *B. besnoiti*-induced NETosis, it is still unclear whether extracellular components (e.g., TLR receptors) exist for the recognition of parasite presence to perform the subsequent NETs-related downstream signaling within the bovine PMN.

### Purinergic Signaling in Suicidal NETosis Against B. besnoiti

Purinergic signaling is among the most primitive and conserved signal transduction systems in evolutionary history [66], mediating the balance between immune cell functions through two major families of purinergic receptors, P2 and P1, which exhibit opposing effects to properly regulate immune responses. Whilst the P2 group members correspond to pro-inflammatory receptors that promote the activation of immune cells, P1 are receptors that restrict that activation. It has been described that the fundamental ligand for P2 purinergic receptors corresponds to extracellular ATP molecules, whose concentrations increase considerably in pathological conditions [67]. When there is stress or damage to the host cells, ATP molecules are released into the extracellular space, which is recognized as a “danger signal” acting as an alarming call for leukocyte recruitment, thereby guiding the migration of PMN, which respond by releasing extracellular ATP and related nucleotides in an autocrine/paracrine manner [68,69].

Purinergic receptors are involved in several essential PMN defense functions, such as chemotaxis [70], phagocytosis, oxidative burst (ROS production), and degranulation [68]. Interestingly, the role of purinergic signaling in NET formation against protozoan parasites has also been reported recently, particularly the group of P2 receptors [71]. Nonetheless, little is known about purinergic pathways in the bovine system. In this regard, *B. besnoiti*-induced NETosis was found to be dependent on the P2X1-mediated purinergic pathway, since a first functional inhibition experiment showed that via blockage of P1A1- and P2X1 purinergic receptors, this defense process selectively depended on P2X1-mediated ATP binding but was independent of P1A1-mediated purinergic signaling [42]. Similarly, another study confirmed that bovine *B. besnoiti*-mediated NETosis was dependent on P2X1 purinergic signaling, further providing evidence of the independence of purinergic receptors P2Y2, P2Y6, P2X4, and P2X7, which failed to influence *B. besnoiti*-driven NETosis after tachyzoite stimulation [44]. In line with this, studies regarding other apicomplexan parasite-related studies have proven the involvement of the P2X1 receptor in *E. bovis*- and *C. parvum*-mediated NETosis [71], since experimental inhibition with NF449 pre-treatment significantly decreased NET formation in parasite-exposed bovine PMN when compared to non-treated controls. Therefore, a conserved P2X1-related signaling pathway has been proposed in bovine NETosis driven by apicomplexan parasites [44]. It is worth mentioning that, through experimental inhibition of the purinergic P2X1 receptor by using the purinergic antagonist NF449, it was demonstrated that NETosis blockage was dose-dependent with respect to the inhibitor, without affecting the cell viability of bovine PMN. Therefore, blocking the P2X1 receptor did not lead to any alternative cell death such as apoptosis or necrosis [44]. Additionally, an interesting finding was the evident clustering of bovine PMN in a P2X1-dependent manner when exposed to *B. besnoiti* tachyzoites over a 4 h time period, reinforcing the relevance of P2X1 in promoting PMN aggregation to NET against this parasite [44]. This PMN clustering may represent an early event in NET formation triggered by *B. besnoiti* tachyzoites in vivo, but further investigations are needed. Interestingly, although NET formation was significantly induced, exposure to *B. besnoiti* tachyzoites did not modify the total intracellular ATP in exposed PMN or the amount of extracellular ATP [44]. Despite this, ATP still plays a key role in purinergic signaling, where P2X receptors function as ATP-gated ion channels, facilitating the entry of extracellular cations, including calcium influx [68]. To date, specific downstream molecules of P2X1-driven purinergic signaling in bovine NET formation triggered by *B. besnoiti* must be further studied to elucidate the connection between the different signaling pathways to perform NETosis in the bovine innate immune system.

## 4. Metabolic Changes Linked to Bovine NETosis Against *B. besnoiti*

Immune cells have been described to undertake metabolic shifting or reprogramming from their resting state towards an activated state to modulate immune function under the concept of “immunometabolism” [72]. Regarding PMN, metabolism has been proposed to be a pivotal element involved in NETosis at different levels [28,42,50,73,74]. For instance, the release of PAF-induced NET from bovine PMN was shown to be dependent on glycolysis, mitochondrial ATP synthesis, and purinergic signaling. Moreover, neutrophils have been described for manifesting metabolic changes after confrontation with apicomplexan parasites [71]. Several studies have focused on the metabolic signaling pathways related to bovine NETosis induced by *B. besnoiti* tachyzoites and bradyzoites [41,42,44,45].

In addition to the description of extracellular ATP-related purinergic activation as a requirement for proper PMN functionality [44], the intracellular origin of ATP that drives these mechanisms is an important piece to elucidate the effector cascade. Overall, mammalian cells produce much of their intracellular ATP by glycolysis, which takes place within the cytosol, or by oxidative phosphorylation and ATP synthesis, both of which are carried out by mitochondria [75]. Mitochondrial-derived ATP molecules have been associated with metabolic changes that activate PMN [73].

In this sense, an important role for ATP regeneration within the mitochondrial respiration chain was indicated during NETosis driven by *B. besnoiti* tachyzoites [42]. This finding was determined by pharmacological inhibition experiments comprising pre-treatment with oligomycin (a mitochondrial ATP synthase inhibitor) in bovine neutrophils for 30 min, followed by exposure to *B. besnoiti* tachyzoites for 3 h. The results revealed a considerable decrease in the quantification of extracellular DNA (considered as the amount of “cell-free” NET) induced by *B. besnoiti* tachyzoites, whose values were even lower than those of the control group (plain neutrophils) [42]. A key role of the mitochondrial ATP-mediated metabolic pathway was indicated in *E. bovis*-triggered NET formation, since a significant decrease in “cell-free” and “anchored” NET release by oligomycin A treatment was observed [71]. Additionally, scanning electron microscopy (SEM) analysis showed that an inhibition pre-treatment with oligomycin on human neutrophils revealed little to no effect on PMA-induced NET formation, suggesting that mitochondrially generated ATP (oxidative phosphorylation) is not required in this process. Surprisingly, PMA-induced NET quantified by ImageJ image processing software analysis was shown to be diminished within the same oligomycin experiment, finally proposing that mitochondria could have a partial role in PMA-stimulated NETosis [74]. Similarly, Bao et al. [75] reported that glycolysis is the main mechanism by which human PMN generate intracellular ATP; however, mitochondria also play an essential role in PMN activation. In brief, ATP contribution is not entirely attributable to the glycolytic pathway but is also partially driven by mitochondria, although these organelles are particularly scarce in mammalian PMN [76].

Conversely, regardless of the ATP source and considering that ATP molecules as well as *B. besnoiti* parasites lead to NET formation, it is intriguing which metabolic parameters are modified by either ATP molecules or *B. besnoiti* tachyzoites to induce suicidal NETosis. In this regard, previous studies using Seahorse^®^ technologies have standardized the analysis of rapid metabolic activities involved in bovine PMN activation exposed to protozoa by measuring oxygen consumption rates (OCR), extracellular acidification rates (ECAR), and linking those parameters to ROS production [71]. Regarding *B. besnoiti*-induced NET formation, the metabolic state of early bovine PMN activation was also evaluated by OCR- and ECAR analyses [44], in order to test the metabolic effects influenced by the presence of either tachyzoites or ATP. Results revealed that exposure to *B. besnoiti* tachyzoites significantly increased OCR in stimulated bovine PMN, without affecting ECAR. Therefore, it was proposed that tachyzoites promote bovine PMN to modify their energy status towards aerobic metabolism, as recently demonstrated [44]. Intriguingly, the OCR measure in co-culture of PMN and vital *B. besnoiti* tachyzoites was carried out, which may have interfered with the general oxygen consumption analysis. Even though, the progressive increase in the values of oxygen consumption registered from *B. besnoiti*-exposed PMN was associated with a probable oxidative burst (ROS production) in bovine PMN [44]. However, because ATP is involved in the metabolic and signaling responses of activated bovine PMN, the effect of supplementation with non-modified ATP was also evaluated. In contrast to the parasite-influenced metabolic effect, treatment of plain bovine PMN with ATP did not affect OCR but instead significantly increased the ECAR rate, which indicates that ATP supplementation shifted PMN into a glycolytic status [44]. Considering that both the parasite and ATP influence cellular metabolism, we investigated whether non-modified ATP molecules exert an additive effect on *B. besnoiti* tachyzoite-induced activation of bovine neutrophils. Thus, a pre-treatment with 50 µM non-modified ATP was added to PMN, which were subsequently exposed to *B. besnoiti* tachyzoites. The results revealed that neither parasite-driven OCR increase nor ECAR basal values were changed by previous ATP supplementation, thereby denying a priming effect of ATP acting as a paracrine signal [44]. Additionally, it was investigated whether a potential change in extracellular ATP values as well as intracellular total neutrophil ATP exists when bovine PMN are exposed to *B. besnoiti* tachyzoites, revealing that neither changes in extracellular ATP- nor total PMN ATP concentrations were detected [44]. Overall, the maintenance of ATP concentrations could be explained by the constant regulation of ectonucleotidase activities, which are enzymes expressed in the plasma membrane of mammalian PMN. Therefore, ATP pre-treatment did not show any effect on NET formation itself or *B. besnoiti* tachyzoite-induced NETosis, in contrast to non-hydrolyzed ATP (ATPγS) pre-treatment, which successfully triggered this effector mechanism and boosted *B. besnoiti* tachyzoite-driven NETosis as well [44].

### Pyruvate- and Lactate-Mediated Metabolic Pathways Are Implicated in B. besnoiti Tachyzoite-Induced NET Formation

In addition to the indicated role of ATP availability above, it was evaluated the involvement of pyruvate, lactate, alanine, aspartate, glucose, serine, glutamine and glutamate to explore the metabolic signatures implicated in bovine NETosis induced by viable *B. besnoiti* tachyzoites [42]. Cytosolic pyruvate originates from several sources (e.g., glycolysis, serine, threonine) and can be directly released into the extracellular medium or after being transformed into lactate or alanine [77,78]. Pharmacological intervention at the level of the glucose-pyruvate-lactate axis confirmed the importance of lactate and pyruvate generation during bovine NETosis induced by *B. besnoiti* tachyzoites, since experimental treatments with both oxamate (inhibitor of lactate dehydrogenase) and dichloroacetate (inhibitor of pyruvate dehydrogenase kinase) efficiently hampered *B. besnoiti*-induced ‘cell-free NETs’, leading to a NET diminishment compared to the non-stimulated PMN control group [42]. A key role of lactate-mediated metabolic pathway was indicated in *E. bovis*-triggered NET formation, since NET release was significantly reduced by oxamate treatments [71], which was also described similarly in PMA- and A23187-induced human NET formation as well as in a mice LPS-induced sepsis model [72]. Alternatively, pyruvate can remain intracellular and enter the Krebs cycle, providing further ATP molecules [78]. Considering this, pre-treatment with oxythiamine (inhibitor of transketolase and tricarboxylic cycle-related enzymes pyruvate dehydrogenase and α-ketoglutarate dehydrogenase) led to a significant reduction in *B. besnoiti*-triggered ‘cell-free NETs’ [42]. In contrast, supernatant analysis of *B. besnoiti* tachyzoite-exposed PMN showed a trend toward increased liberation of pyruvate, lactate, and alanine, in addition to an increased rate of aspartate consumption, none of which reached significant levels in their metabolic changes [42]. Nonetheless, a significant increase in glucose consumption directly attributable to bovine PMN was observed, confirming the utilization of glucose within the metabolic signatures. Nevertheless, functional inhibition of glycolysis at the hexokinase level via the inhibitor fluoro-2-deoxy-D-glucose (FDG) did not affect *B. besnoiti*-induced NET formation [42]. Thus, it was proposed that the glycolytic pathway may not have influence in bovine NETosis triggered by this species so far, although glucose consumption was significantly involved of note. Conversely, PMA-induced NET formation in human PMN was shown to be dependent on the presence of extracellular glucose; however, contrary to the findings of Zhou et al. [42], it was suggested that human NETosis is a glycolysis-dependent process (specifically the last stage for the release of NET) in which mitochondria-derived ATP by oxidative phosphorylation is not practically required [74]. Additionally, a significant increase in serine consumption was observed after bovine PMN were confronted with *B. besnoiti* tachyzoites, which may explain pyruvate release, as serine is a source of pyruvate [42,77]. Interestingly, another amino acid measured in the same supernatant analysis was glutamine, which was produced in similar amounts in both *B. besnoiti*-exposed and non-exposed PMN. Therefore, this metabolite was not indicated as an energy source in such immune cells [42]. The significant increase in glutamate release could explain the decreased output of glutamine in *B. besnoiti*-confronted PMN, but this requires final clarification [42].

Taken together, the data point toward the utilization of glucose and serine, and their consumption results in greater regenerative energy conversion to pyruvate and lactate. Therefore, pyruvate- and lactate-mediated metabolic pathways are key players in *B. besnoiti* tachyzoite-triggered NETosis [42]. In other words, the secondary metabolites of carbohydrate catabolism, rather than glycolysis itself, may play a pivotal role in *B. besnoiti*-NET formation in the bovine system.

## 5. Autophagy-Related Signaling and *B. besnoiti*-Induced Bovine NETosis

Autophagy represents an essential intracellular degradation process that recycles cellular components (e.g., damaged organelles and proteins) by transporting cytoplasmic material to lysosomes or autophagic vesicles for degradation. This process is crucial in a wide range of physiological and pathological conditions since it participates in cellular responses to stress events [79]. Indeed, autophagy has been attributed to a regulatory role in the induction of NETosis, since autophagy activation (via inhibition of the mTOR pathway or activation of cGAS-STING signaling pathway) has been reported to promote NET formation, whereas autophagy inhibition, by using 3-methyladenine (3-MA) (blocks phosphatidylinositol 3-kinase (PI3K) activity) or PtdIns3K inhibitor, reflected a decrease in NET release [80,81]. In addition, NETosis and autophagy are interconnected processes in ROS-dependent and PMA-triggered NET formation in human PMN [82]. AMP-activated kinase α (AMPKα), one of the most studied autophagy-related enzymes, has also been identified as an important metabolic sensor molecule in the regulation of several PMN processes, such as glycolytic metabolism [83], ROS production [84], chemotaxis [85], phagocytosis [86], and well as in the formation of NETs [87,88]. Autophagy-related experiments have been addressed in the field of parasite-induced NETs, including *B. besnoiti* [41,45]. A positive correlation between autophagy and *B. besnoiti*-triggered suicidal NETosis has been found, as both NET and autophagosome formation occurred simultaneously in exposed bovine PMN to vital motile tachyzoites [41]. Notably, the microtubule-associated protein LC3 is a key marker of autophagic activity [80]. In mammals, LC3-I is conjugated to phosphatidylethanolamine (PE) to form LC3-II during autophagy, and thus, it is useful for detecting autophagy activity. For instance, a significantly increased LC3-II/LC3-I ratio has been observed in PMN during *P. plecoglossicida* infection compared to controls [81]. Regarding *B. besnoiti*, it was observed that PMN exposure to tachyzoites led to significant autophagosome formation evidenced by LC3B expression. Interestingly, LC3B-stained autophagosomes were concomitantly detected in PMN extruding suicidal NETosis against the chronic phase-related *B. besnoiti* bradyzoites [43] as well, which indicates that autophagy seems to be a parasite stage-independent mechanism performed during *B. besnoiti*-induced NETosis.

Moreover, both autophagy and *B. besnoiti*-induced NETosis were associated with rapid phosphorylation of AMPKα within the first 5–30 min of parasite exposure [41,45]. Therefore, AMPK seems to be relevant in the early process of *B. besnoiti*-triggered NETosis, which was also confirmed through AICAR treatment in bovine PMN, showing significant enhancement compared to plain parasite-induced NET release [45]. Notably, AICAR is an autophagy stimulator that phosphorylates AMPK in human PMN [84]. Complementarily, the role of AMPK was confirmed in the early step of *T. gondii*-mediated NETosis, since AICAR pre-treated PMN and then exposed to *T. gondii* showed a significant increase in NET formation when compared to the groups of sole parasite exposure and non-exposed bovine PMN [89]. Furthermore, Western blot (WB) analysis revealed that phosphorylated AMPK (pAMPK), but not total AMPK, significantly enhanced its expression after 30 min of co-culture of PMN with *T. gondii* [89]. Notably, AMPK is a serine/threonine protein kinase complex consisting of a catalytic α-subunit (α1 and α2), a scaffolding β-subunit (β1 and β2), and a regulatory γ-subunit (γ1, γ2, and γ3) [90]. Investigation on AMPK subunits activation revealed that despite no changes observed for the regulatory subunits AMPKβ1 and AMPKγ1 in *B. besnoiti*-exposed PMN, there was indeed a moderately enhancement of catalytic subunit AMPKα1 expression but still statistically insignificant [45]. AMPK activation induces autophagy through two different downstream mechanisms. First, AMPK negatively regulates the mammalian target of rapamycin (mTOR) protein kinase complex; second, AMPK activates Unc-51-Like Kinase 1 (ULK1) by direct phosphorylation [91,92]. Moreover, ULK1 can induce autophagy by phosphorylating Beclin-1 protein [93]. Exposure of bovine PMN to *B. besnoiti* tachyzoites (at a 1:6 ratio) showed a significant upregulation of ULK1 after 30 min of co-incubation, which temporally coincided with AMPK activation [45]. Nonetheless, Beclin-1 expression and its phosphorylated form (p-Beclin-1) reached values similar to those of the control group [45]. Recent kinetic investigations on concomitant autophagy and *T. gondii*-induced bovine NETs also revealed that phosphorylated and unphosphorylated ULK-1 units were upregulated; however, neither form reached statistically significant increases [89]. Interestingly, autophagy related to *B. besnoiti*-induced NETs seems to be independent of both the mammalian target of rapamycin (mTOR) and phosphatidylinositol 3-kinase (PI3K) signaling pathways, since pharmacological treatments with their respective inhibitors (rapamycin and wortmannin) did not affect *B. besnoiti*-induced NET formation [41]. Notably, the mTOR signaling pathway has been shown as a pivotal node in autophagy regulation during human NETosis, since inhibition of mTOR signaling axis promoted PMN autophagy and accelerating formation of human-derived NETs [80,88]. Moreover, other autophagy inhibitors, such as PI3K (LY294002), NF-κB (pathenolide), and deubiquitinase (WP1130), failed to affect *B. besnoiti*-triggered NETosis, discarding the participation of PI3K, NF-κB, and deubiquitinase in this cell death process [41].

On the other hand, AMPK activity is regulated by upstream kinases such as CAMKK (Ca^2+^/calmodulin-dependent protein kinase) [90]. Consequently, WB analyses revealed that both phosphorylated and non-phosphorylated CAMKK were significantly upregulated in bovine PMN immediately after 5 min of *B. besnoiti* tachyzoite encounter [45]. Similarly, it has been reported that both phosphorylated and non-phosphorylated CAMKK were upregulated in *T. gondii*-exposed PMN after 30 min of co-incubation, indicating sustained activation of CAMKK in *T. gondii*-induced NET formation [89]. Notably, fluorescence changes have evidenced a rapid and significant increase in PMN intracellular calcium concentration [Ca^2+^]_i_ after 5 min of co-incubation of PMN with *T. gondii*, when compared to negative controls (plain PMN) [89]. Previous studies have shown that *T. gondii*-induced DNA release depends on store-operated calcium entry (SOCE), as pharmacological blockage significantly diminishes extDNA counts from exposed bovine PMN [89]. Considering the involvement of CAMKK- and SOCE signaling linked to apicomplexan-induced DNA extrusion of bovine PMN [45,89], calcium influx seems to have an interesting role in molecular signaling, allowing early innate defense mechanisms in cattle. Nevertheless, further studies are needed to elucidate the signaling functions of calcium flux in the bovine system, particularly in *B. besnoiti*-induced NETosis.

## 6. Interaction Between Bovine NET and Endothelium in the *B. besnoiti*-Generated Infection

Vascular and lymphatic endothelial cells are the main targets for the replication of *B. besnoiti* tachyzoites, which rapidly proliferate during the acute phase of bovine besnoitiosis in vivo [22,24,94,95]. Additionally, due to the close interaction between pathogens, active immune cells and endothelium, effects of NET release on *B. besnoiti*-infected bovine endothelium have been questioned. The endothelium is a monolayer comprising cells with immunoregulatory functions that form part of several dynamic processes to combat pathogens [65,96]. RT-qPCR analysis demonstrated that *B. besnoiti*-infected primary bovine umbilical vein endothelial cells (BUVEC) promptly reacted by producing significantly immunoregulatory molecules such as CXCL1, CXCL8, CCL5, and COX-2 [40]. Additionally, increased adhesion molecule expression (e.g., ICAM1) led to PMN rolling and adhesion to *B. besnoiti*-infected BUVEC [40]. Overall, data indicate that *B. besnoiti*-infected endothelium triggers a cascade of pro-inflammatory reactions leading to endothelial cell activation by increasing the release of immunoregulatory molecules and upregulating adhesion molecules, which enhances PMN adhesion [40,97].

It is worth mentioning that PMN adhesion is overall mediated through surface receptors interacting with specific ligands presented on surfaces [98]. Integrin ligands have been shown to play an important role in leukocyte adhesion and migration [98], indicating that PMN adhesion is managed by a complex network of combined processes and molecules. For instance, the chemokine IL-8 is a well-known chemoattractant for PMN and has been demonstrated to be a NET inducer [30]. During a *B. besnoiti* infection, once PMN are attached to the infected endothelium, they can perform NET extrusion, which has been evidenced by DNA staining and SEM analysis [40]. In agreement with these findings, bovine PMN have also been found to undergo ‘anchored-NET formation’ on *B. besnoiti*-infected BUVEC under physiological flow conditions, which was corroborated by the co-localization of extracellular DNA decorated with histones [41]. PMN have been described as being associated with pathophysiological conditions, since excessive quantities of released NET seem to contribute to collateral tissue damage [28,99,100]. Excessive release of NET has been shown to affect the endothelium by causing increased permeability and/or directly damaging individual endothelial cells [101]. In particular, the contribution of citrullinated histones to NET-derived pathogenic effects on individual cells and tissues has been well described [28]. Notably, extracellular histones can act as DAMPs, activating several signaling pathways [65]. Histones, including H1, H2A, H2B, H3, and H4, are the major constituents of released NETs, accounting for approximately 70% of all NET proteins [36,102], in which H2A represents 26.9% of the total NET protein content [103] and is also described as one of the main inducers of cytotoxicity [101]. Remarkably, differences in cytotoxicity depend on the type of histones, with H2A, H2B, and H4 being individually more cytotoxic than a mixture of the other histones [104]. Regarding bovine besnoitiosis, Conejeros et al. [26] proposed a pathophysiological relevance of NET-derived histones in tissue damage during *B. besnoiti* infections, since treatments with isolated H2A (200 µg/mL) from released *B. besnoiti*-triggered NET significantly induced cytotoxicity and damage on non-infected BUVEC when applied for 4 or 12 h, compared to the non-treated control group. Furthermore, pure *B. besnoiti* tachyzoite-stimulated NET preparation at a concentration of 3.3 ng DNA/mL also induced significant cytotoxic effects on non-infected BUVEC after 12 h of exposure in a static endothelium system [26]. To be as close to in vivo situation of cattle besnoitiosis, a constant physiological shear stress system was designed consisting in a 5 min period of PMN or medium alone perfusion over non-infected BUVEC and *B. besnoiti*-infected at 12 h post-infection (p.i.) [26]. Results revealed that PMN perfusion on non-infected BUVEC induced an average damage of 17.48%, whereas the control group of sole medium perfusion barely affected non-infected BUVEC layers, of which 2.83% were damaged on average [26]. Moreover, once bovine PMN were perfused onto *B. besnoiti*-infected endothelial cells, cell damage increased to 35.47% at 12 h p.i., whereas the group with medium perfusion to *B. besnoiti*-infected BUVEC did not induce considerable cell damage, as only 3.43% of endothelial cells were affected [26]. Notably, by comparing both sole medium perfusion-related groups, measurements of cytotoxicity on non-infected and parasite-infected BUVEC did not differ significantly [26]. Despite the negative effect on host endothelial cells, *B. besnoiti*-induced NET treatments did not significantly affect the total proliferation of *B. besnoiti* tachyzoites from infected BUVEC measured at 30 h p.i., although the diameter and number of parasitophorous vacuoles (PV) per infected endothelial cells diminished after NET treatments at 12 h p.i. [26]. Therefore, a direct effect of parasite-triggered NET on the intracellular replication of *B. besnoiti* was denied, since NET did not interfere with overall parasite proliferation.

Considering all the data, Conejeros et al. [26] postulated that excessive NET formation may contribute to pathogenesis driven by toxic side effects on cells and that these immune defense-related structures do not exert lethal effects on tachyzoite stages. Additionally, it is not clear how NETs are indirectly triggered by tachyzoites when parasites are inside host cells. Thus, further studies are needed to elucidate whether host endothelial cells express parasite-derived antigens on their surface membrane to be eventually recognized by PMN-associated pathogen-recognition receptors (PRRs) and then induce NET formation.

## 7. *Besnoitia bensoiti* Bradyzoites Trigger Suicidal and Vital NETosis

**Figure 1:**
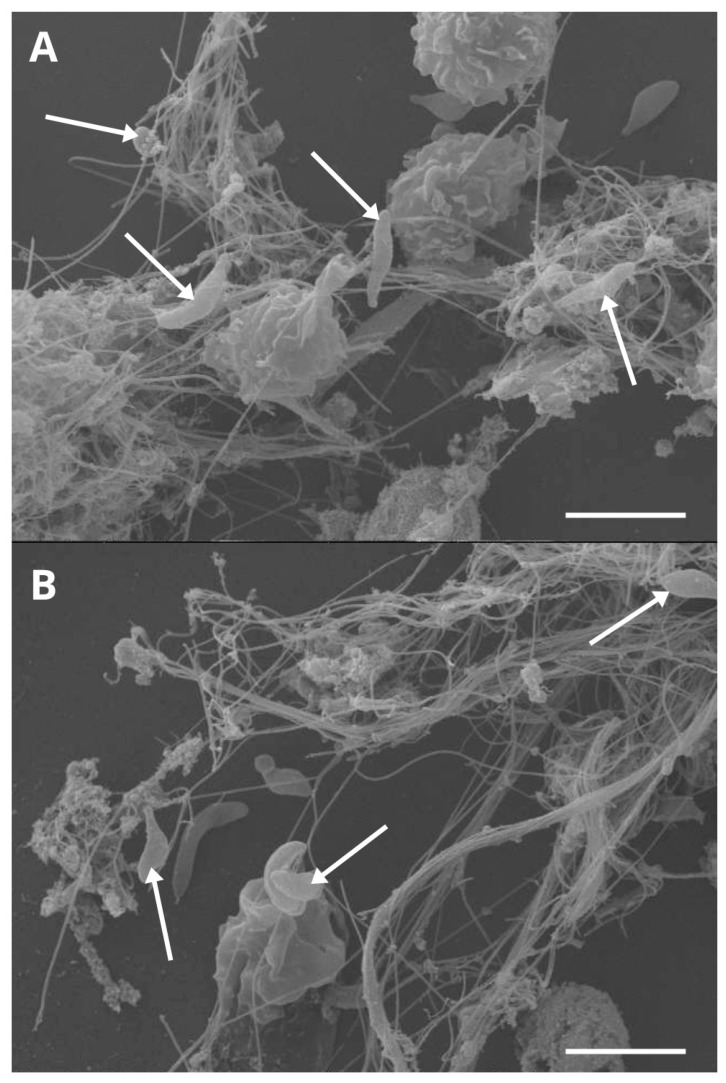
Bovine NETosis after confrontation with *Besnoitia besnoiti* bradyzoites (**A**,**B**). Scanning electron microscopy (SEM) analysis unveiled release of NETs being formed by bovine PMN co-cultured with viable *B. besnoiti* bradyzoites, and these extracellular fibers resulted in a fine meshwork containing bradyzoites as indicated by white arrows. Scale bar = 5 µm.

In addition to suicidal NET formation, which is an event that requires lytic death of cells, neutrophils have also been shown to undergo vital NET formation that occurs faster and without cell lysis, thereby remaining viable with intact nuclear membranes and preserving the ability of active phagocytosis of bacteria [53,105]. In vital NET formation, NETs are released through intracellular vesicles containing DNA that fuse with the outer membrane, resulting in the delivery of NETs to the extracellular space without cell lysis [105]. At first glance, suicidal NETosis has been reported once bovine neutrophils were exposed to motile *B. besnoiti* bradyzoites entrapping firmly parasites by cell death, which was visualized through scanning electron microscopy (SEM) analysis [43] as illustrated in Figure 1. Classical components of NETs were demonstrated by the co-localization of extracellular DNA adorned with histone H3 and NE [43]. Furthermore, a rapid vital NETosis was observed against *B. besnoiti* bradyzoites within the first 30 min of exposure, which occurred without compromising the overall structure of the PMN cell membrane [43]. Using live cell 3D holotomographic microscopy, vital NETosis mediated by *B. besnoiti* bradyzoites showed initial pseudopod formation on bovine neutrophils, and the outcome was described as a non-lytic rapid extrusion and retraction of a ‘chameleon tongue’-like structure after 30 min of parasite interaction, which is the first allusion of this type of NETosis against apicomplexan parasites [43]. Vital NET formation has been described as an oxidant-independent mechanism in human PMN [53]. Nevertheless, it remains unclear which signaling pathways are involved in the machinery to perform NET formation without compromising the general structure of the neutrophil cell membrane in the bovine system [43]. To date, it has been suggested that this process can be the first effector mechanism that occurs against invasive pathogens [38], but it has not been determined how vital NETosis might contribute to infection control. It is proposed that, although bradyzoites are stored in tissue cysts, the eventual rupture of these cysts located directly beneath the vascular endothelium would allow the entry of bradyzoites into the bloodstream, constituting a possible scenario in which PMN would be able to release NET in response to these bradyzoites, as demonstrated experimentally [43]. Finally, it can be suggested that NET formation triggered by *B. besnoiti* is a stage-independent process, as the development of NETosis against tachyzoites [24] and bradyzoites [43] has been described.

## 8. Conclusions

Active infection with *B. besnoiti* tachyzoites and bradyzoites strongly induced the release of bovine NETs, corresponding to suicidal NETosis. *B. besnoiti*-induced NET formation is a mechanism that involves NOX-derived ROS production and the enzyme activities of NE and MPO. Concerning antiparasitic effects, cast NETs can immobilize but not kill tachyzoites; nonetheless, these structures result in reduced obligate host cell invasion. *B. besnoiti*-triggered NETosis shows a dose-dependency; however, it is a parasite stage-independent process being able to firmly entrap tachyzoites and bradyzoites as well. Moreover, bovine NETs are stimulated independently of the exposure of different integrity or viability status of *B. besnoiti*. Signaling pathways involved in *B. besnoiti*-induced NETosis point to a dependency on the P2X1 receptor-mediated purinergic pathway, and the key relevance of ATP availability for PMN activation via autocrine/paracrine signaling has been proposed. Notably, *B. besnoiti*-induced ‘cell-free NETs’ seem to depend on mitochondrial activities but not on glycolysis. Regarding metabolic pathways, *B. besnoiti*-mediated NET formation is linked to pyruvate and lactate catabolism, as well as AMPK axis activation. Both stages, tachyzoites and bradyzoites, stimulate PMN to simultaneously perform autophagy and NET formation; however, both processes are independent of each other. Bovine NET formation damages the *B. besnoiti*-infected endothelium, mainly due to the cytotoxic activity of H2A, which is abundantly present in these extracellular chromatin networks. To the best of our knowledge, this is the first review specifically focused on summarizing the existing evidence regarding signaling pathways and cellular metabolism in bovine neutrophils involved in the formation and release of DNA extracellular traps against the parasite *B. besnoiti*, whose infection is currently spreading in different regions of the world, particularly in countries with traditional livestock farming. In addition, this review provides new perspectives on the NETosis mechanism—specifically addressed here within the bovine system—which has recently been investigated as a potential therapeutic target due to its described pathophysiological implications in well-known human viral (e.g., COVID-19), autoimmune, and neoplastic diseases. Regarding bovine besnoitiosis, given that *B. besnoiti* causes systemic and localized lesions in cattle, constituting a potential threat of economic losses in production systems through reduced production/fertility and treatment costs, it underlines the importance of understanding how excessive NETs extrusion might contribute to pathogenesis, as reported for other NET-associated vascular- (atherosclerosis, atherothrombosis) and epidermal disorders [psoriasis, systemic lupus erythematosus (SLE)]. Therefore, further studies are needed to elucidate the implications of NET-derived adverse effects in vivo for eventual therapeutic applications, as there are currently no effective treatments for bovine besnoitiosis.

## Figures and Tables

**Table 1:** Main scientific publications selected by inclusion criteria.

Authors (Year)	Host Species/ Cell Type	Parasite Stage	Experimental Approach	Main Findings
Muñoz-Caro et al. (2014) [24]	Bovine neutrophils	Tachyzoites	Staining and antibodies for detection of NETs compounds. Spectrofluorometric analysis for quantification of NETs. Treatments with DNase I and inhibitors of NOX, NE and MPO.	- *B. besnoiti* tachyzoites rapidly trigger NET release in bovine neutrophils. Bovine NETs co-localize with histones, NE, and MPO, and their formation is reduced by DNase I or NOX/NE/MPO inhibition. NETs immobilize parasites and decrease host cell invasion.
Maksimov et al. (2016) [40]	Bovine neutrophils, BUVEC	Tachyzoites	PMN adhesion assay under physiological flow conditions.	- *B. besnoiti* infection leads to endothelial cell activation which increased gene transcriptions of molecules with immunoregulatory functions and enhanced levels of PMN adhesion being accompanied by upregulated adhesion molecule gene transcription.
Conejeros et al. (2019) [26]	Bovine neutrophils, BUVEC	Tachyzoites	Confocal and fluorescence microscopy for detection of extracellular DNA and protein markers of NETs. Fluorescence analysis for estimation of NET-, DNA- and histone 2A (H2A)-induced endothelial cell death. Protein isolectin GS-IB4.	- Evidence showing that *B. besnoiti* tachyzoite- and A23187-induced NETs, as well as histone H2A, exert damaging effects on host cells. - NETs induced by *B. besnoiti* tachyzoites do not influence the total parasite proliferation on infected primary endothelial cells.
Zhou et al. (2019) [41]	Bovine neutrophils	Tachyzoites	“Cell-free”-NETs and “anchored”-NETs estimation. Immunofluorescence analysis. Immunoblotting-based analysis.	- Confrontation of PMN with *B. besnoiti* tachyzoites clearly induced AMPKα activation in a time-dependent manner. - AMPKα activation (via phosphorylation) occurred rapidly after parasite–PMN contact and lasted up to 30 min.
Zhou et al. (2020) [42]	Bovine neutrophils	Tachyzoites	Estimation of metabolic conversion rates through pharmacological inhibition and supernatant analysis.	- Highlights the role of metabolic pathways, purinergic signaling, and pH conditions in *B. besnoiti* tachyzoite-induced NETosis in bovine neutrophils.
Zhou et al. (2020) [43]	Bovine neutrophils	Bradyzoites	Histopathological examination. Immunofluorescence microscopy analysis. Live cell 3d holotomographic microscopy.	- First description of bovine PMNs releasing NETs against motile *B. besnoiti* bradyzoites. - Autophagy (LC3B-positive) correlates with suicidal NETosis, suggesting interplay between both pathways; also reports vital NETosis characterized by rapid extrusion–retraction (“chameleon tongue-like”) structures.
Espinosa et al. (2023) [44]	Bovine neutrophils	Tachyzoites	Immunofluorescence microscopy. Picogreen-derived fluorescence intensities. Luminometry. Seahorse XF analyzer. Flow cytometry.	- Reveals P2X1-mediated purinergic signaling as a key factor in *B. besnoiti*–PMN interactions, extending its role beyond NETosis to additional PMN effector mechanisms.
Conejeros et al. (2024) [45]	Bovine neutrophils	Tachyzoites	Protein Extraction and Western blot. Seahorse XF analyzer. Flow cytometry. Immunofluorescence.	- AICAR alone treatment induced NET formation with mitochondrial and glycolytic activation in bovine PMNs; showed additive effects with *B. besnoiti* tachyzoites and moderate NOX-dependent oxidative responses.

## Data Availability

The authors wish to disclose that this article includes no new data. All data referenced in this study are derived from the existing literature and publicly available sources. No additional data sets or materials are provided beyond those discussed in the article.

## Abbreviations

The following abbreviations are used in this manuscript:

EFSA	European Food Safety Authority
ETs	Extracellular traps
MPO	Myeloperoxidase
NE	Neutrophil elastase
NETs	Neutrophil extracellular traps
NOX	Nicotinamide adenine dinucleotide phosphate oxidase
PMN	Polymorphonuclear neutrophils
ROS	Reactive oxygen species
